# 3D printed rodent skin-skull-brain model: A novel animal-free approach for neurosurgical training

**DOI:** 10.1371/journal.pone.0253477

**Published:** 2021-06-23

**Authors:** Marie Bainier, Arel Su, Roger L. Redondo

**Affiliations:** 1 Roche Pharmaceutical Research and Early Development (pRED), Neuroscience and Rare Diseases, Roche Innovation Center Basel, F. Hoffmann-La Roche Ltd, Basel, Switzerland; 2 Roche Pharmaceutical Research and Early Development (pRED), Pharmaceutical Sciences, Roche Innovation Center Basel, F. Hoffmann-La Roche Ltd, Basel, Switzerland; Virginia Commonwealth University, UNITED STATES

## Abstract

In neuroscience, stereotactic brain surgery is a standard yet challenging technique for which laboratory and veterinary personnel must be sufficiently and properly trained. There is currently no animal-free training option for neurosurgeries; stereotactic techniques are learned and practiced on dead animals. Here we have used three-dimensional (3D) printing technologies to create rat and mouse skin-skull-brain models, specifically conceived for rodent stereotaxic surgery training. We used 3D models obtained from microCT pictures and printed them using materials that would provide the most accurate haptic feedback for each model—PC-ABS material for the rat and Durable resin for the mouse. We filled the skulls with Polyurethane expanding foam to mimic the brain. In order to simulate rodent skin, we added a rectangular 1mm thick clear silicone sheet on the skull. Ten qualified rodent neurosurgeons then performed a variety of stereotaxic surgeries on these rat and mouse 3D printed models. Participants evaluated models fidelity compared to cadaveric skulls and their appropriateness for educational use. The 3D printed rat and mouse skin-skull-brain models received an overwhelmingly positive response. They were perceived as very realistic, and considered an excellent alternative to cadaveric skulls for training purposes. They can be made rapidly and at low cost. Our real-size 3D printed replicas could enable cost- and time-efficient, animal-free neurosurgery training. They can be absolute replacements for stereotaxic surgery techniques practice including but not limited to craniotomies, screw placement, brain injections, implantations and cement applications. This project is a significant step forward in implementing the replacement, reduction, and refinement (3Rs) principles to animal experimentation. These 3D printed models could lead the way to the complete replacement of live animals for stereotaxic surgery training in laboratories and veterinary studies.

## Introduction

Studying rodent brains, their circuits and the effects of therapies on these circuits is a critical part of drug development for neurological disorders.

Stereotaxic brain surgery is a common procedure routinely performed to this end in neuroscience research [1]. However, stereotaxic surgery remains a challenging technique requiring high technical and manual skills for which surgeons need extensive training.

Proper surgical techniques are fundamental for animal welfare and for high-quality science [2]. From viral injections to long-term implantations, improper surgery techniques could lead to tissue damage, infections, and animal distress. Moreover, for injections and implantations, an inaccurate targeting or placement will lead to inadequate data collection thus making the animal not usable.

Microsurgery skills improve with frequent practice and experience requiring extensive and lengthy training before safely performing stereotaxic surgeries on live animals. The learning process includes a few techniques that can be overwhelming such as anesthesia, sterile work, craniotomies, suturing, etc.

At present, surgeries are typically learned and practiced on dead animals. Besides questions relating to limited animal numbers and availability, the use of animals for this training raises ethical concerns; should animal euthanasia for training individuals be allowed? Unfortunately, to the best of our knowledge there is no current animal-free option to train on rodent stereotaxic surgeries.

While 3D printing technologies have been used extensively in human healthcare for a wide variety of applications: surgery training [3], simulation with patient-specific anatomy reproduction [4], patient-tailored implant/prosthetic production [5], etc., to the best of our knowledge, this technology has never been translated to animal research. In humans, realistic 3D printed models have been successfully used in surgical training for trainees to practice and master relevant procedures [6]. Furthermore, the skills acquired by simulation-based training have been shown to transfer to the operative setting [7].

In the spirit of the 3Rs (Replacement, Reduction and Refinement) we wanted to provide a new method enabling hands-on training of surgeons while improving animal welfare. For that, we developed and validated a new animal-free neurosurgical training model using 3D printing technologies. These 3D printed real-size skin-skull-brain models are meant to enable training on all different types of brain surgeries for both rats and mice.

## Materials and methods

### 1. Skull models

**Fig 1:**
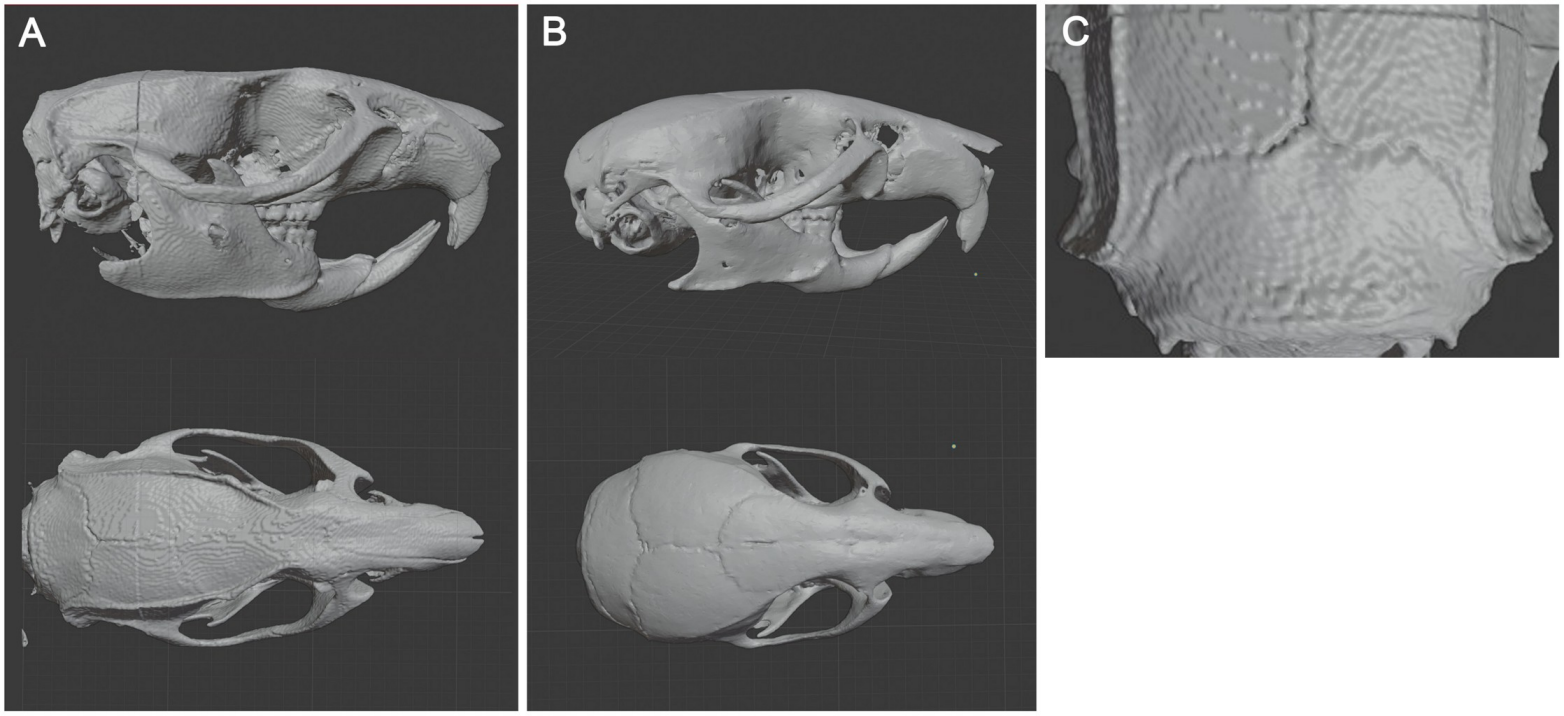
3D models of rat and mouse skull. (A) Rat skull model. (B) Mouse skull model. (C) Close up of landmarks.

Rat [8] and Mouse [9] 3D skull models made from microCT pictures were used in stl format (Fig 1). The process used, from anatomical imaging data to 3D models, has been described step by step by Bernd M Pohl and his colleagues [10].

Both models have been optimized (surface optimization, quality check) using Insight 3D printing software (Stratasys Ltd, USA). Vertebras were removed from the rat 3D model and landmarks made more visible using a dot at the intersections. Size of 3D models can be adapted to the size of the animals used.

Rat skulls were printed using Fortus 380mc FDM (Fused Deposition Modeling) Printer nozzle T10 (Stratasys Ltd, Eden Prairie, MN, USA) with 0.1270mm slice height. The choice of material was oriented to the PC-ABS, which provides better rendering and better haptic feedback, thanks to its flexibility. Black PC-ABS was used to get a better visualization of white dental/bone cement finishes used for long term implantation.

Mice skulls were printed using Formlabs Form3 LFS (Low Force Stereolithography) printer (Formlabs Inc., Somerville, MA, USA) due to the extremely small details of the model. The skulls were printed out of Durable Resin (Formlabs Inc., Somerville, MA, USA) with a 0.05mm layer thickness. After printing, 3D printed skulls were washed with isopropyl alcohol for 10 minutes using a Form Wash machine (Formlabs Inc., Somerville, MA, USA) and cured for 60 minutes at 60°C using a Form Cure machine (Formlabs Inc., Somerville, MA, USA).

Durable Resin is capable of extreme deformation, thus reproducing the mouse skull’s high flexibility. This material only exists in a clear color. The model can be painted (Montana Gold, Montana Cans, Germany) afterward to get a black finish.

For better stability during the printing process, a support material was added and manually removed at the end.

As the mandibles are fixed for both models, lower incisors teeth were cut after printing to enable fixation on a stereotaxic frame.

### 2. Brain model

**Fig 2:**
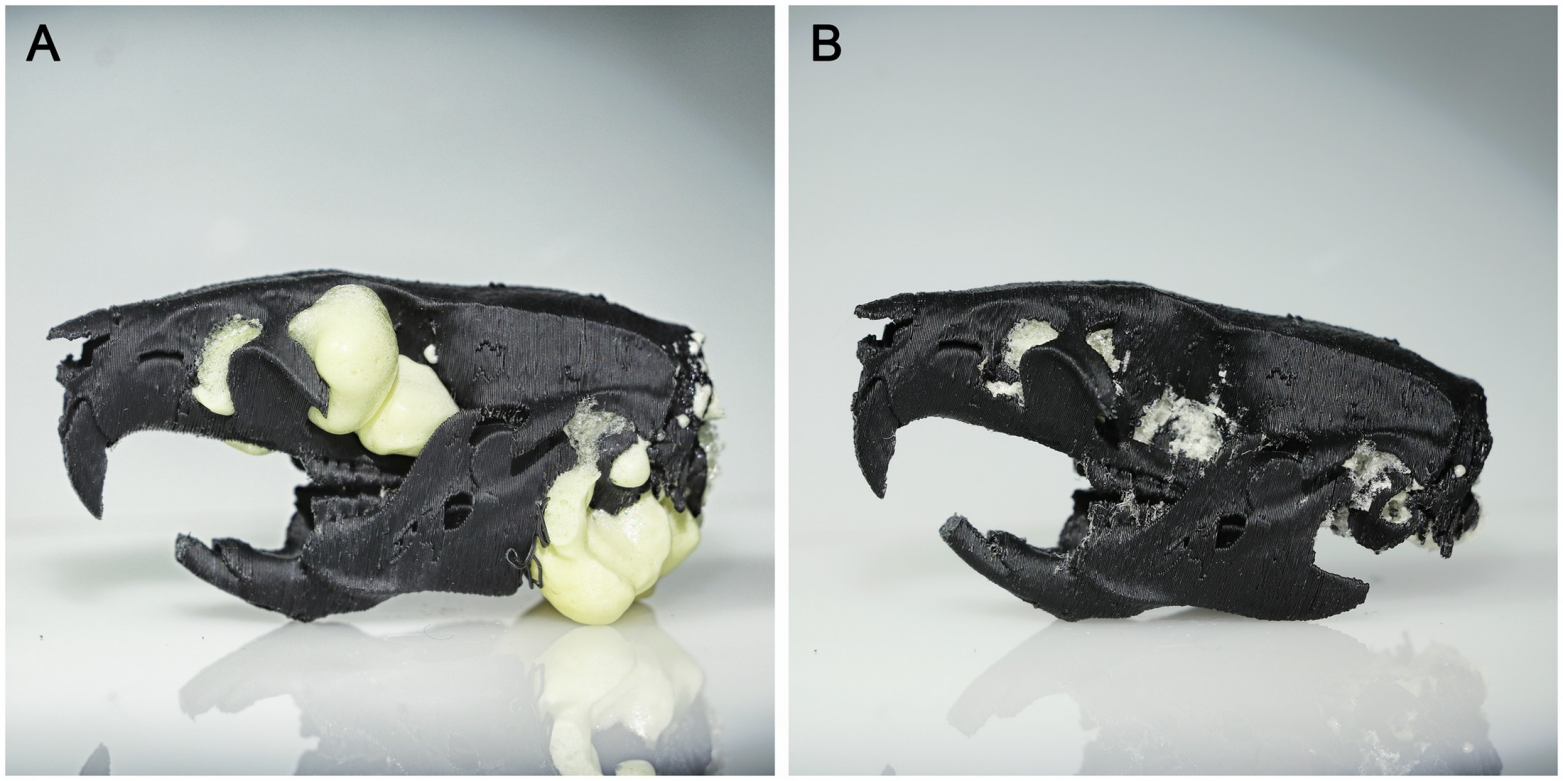
Polyurethane brain manufacturing process. (A) Brain model once dry. (B) Excess of PU foam removed.

The brain was made out of Polyurethane expanding foam (PU 500, Fischer Deutschland Vertriebs GmbH, Germany). The white color of the material makes it possible to see dyes to train on viral injections. Foam was projected from the foramen magnum and was gently filling the whole plastic skull model (Fig 2A). Once fully dry, the foam excess was removed using forceps (Fig 2B).

### 3. Skin model

**Fig 3:**
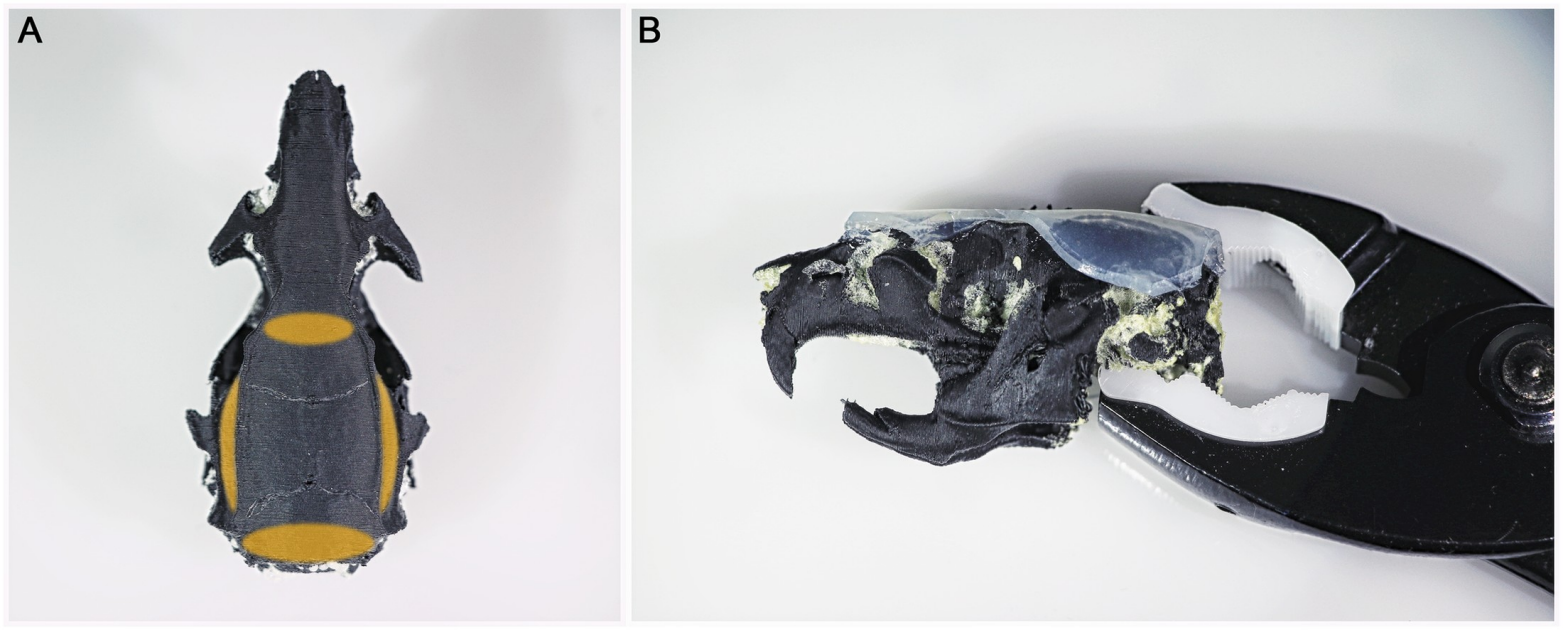
Skin addition process. (A) Zones where glue should be applied. (B) Clamping during the setting time.

To simulate rodent skin a rectangular 1mm thick clear silicone sheet (Polymax, UK) was used. It was secured on the skull using silicon glue (Dowsil^™^ 732, Dow, USA) under both temporal crests, on the lower end of the interparietal bone and on the very front of frontal bone to keep the skull clean of glue over bregma and lambda landmarks (Fig 3A). A clamp was used to keep constant pressure during the curing time (Fig 3B). Silicon was then cut to fit the skull. Skin addition is a long process as one has to glue the silicone on one side after the other with long curing time. We advise adding silicone skin to the model only for suturing training purposes.

### 4. Model validation

We invited ten staff neurosurgeons from Roche to evaluate and test various surgical procedures on the 3D printed skin-skull-brain models. Experience level in stereotaxic surgery varied between participants, from students to experienced scientists.

The skin-skull-brain model was fixed in the stereotaxic frame using teeth and ear bars, as usually done during a live animal procedure. A wide range of neurosurgery techniques, listed below, were successfully tested using our 3D printed models.

#### Craniotomies

These constitute the first step of every brain surgery. They involve the fixation of the skull into the stereotaxic frame, the identification of skull landmarks (bregma, lambda), the positioning of the drill on the target area and the careful drilling and removal of bone tissue. Detailed rodent craniotomies protocol have been described previously [11].

#### Screw placement

A proper positioning of screws in the craniotomy area is essential to achieve good screw stability, in turn allowing screws to work as anchors for potential implants. It is important to set these screws at a reproducible depth, to ensure good data acquisition, e.g. for EEG screws [12].

#### Optic fiber and large Electrophysiology probe implantation and securing using bone/dental cement

A wide range of probes can be implanted in rodents for different functions (brain stimulation, electrophysiology recordings, etc). For acute and long-term implantation, implants need to be firmly secured using dental cement on the skull couple to anchoring screws. Several implantation methods are available, from optic fiber [13] to chronic neural electrode implantations [14, 15].

#### Brain injections

Successful brain injections require a very precise positioning of the needle to target specific brain regions. Identification of brain landmarks and identification of the right coordinates are crucial to ensure that the area of interest is appropriately targeted [16].

#### Suturing

Good sterile suturing techniques are essential to avoid wound dehiscence and infection. Suture pattern and suture material play an important role in wound healing [17].

Surgeons were invited to evaluate the models using anonymous questionnaires. Informed consent was implied if surgeons decided to participate. Participants were surveyed for the models’ fidelity (Table 1) and their usefulness as training tools (Table 2). All responses were recorded using a Likert scale.

**Table 1:** Outline of survey questions: Model fidelity.

Model fidelity	
Questions	Q1: Rate anatomical accuracy
	Q2: Rate model size accuracy
	Q3: Rate landmarks visibility
	Q4: Rate tactile fidelity during surgery (Haptic feedback)
Rating	1: Poor
	2: Fair
	3: Satisfactory
	4: Very good
	5: Excellent

**Table 2:** Outline of survey questions: Appropriateness for educational use.

Appropriateness for educational use	
Questions	Q5: Are these models appropriate and useful for surgery training?
	Q6: Are these models good alternatives to cadaveric skulls for training purposes?
	Q7: Would these models help trainees to develop their confidence?
	Q8: Would you use these models to train someone or to be trained on new techniques?
	Q9: Would you use these models to test the feasibility of new surgeries?
Rating	1: Strongly disagree
	2: Disagree
	3: Neutral
	4: Agree
	5: Strongly agree

## Results

Each 3D printed skull required around 8 hours of non-supervised printing time and a cost of 21 CHF for rat against 2 hours and 5 CHF for mouse. Brain assembly takes a few minutes while the skin addition process (not including setting time) requires less than 20 minutes per skull.

**Fig 4:**
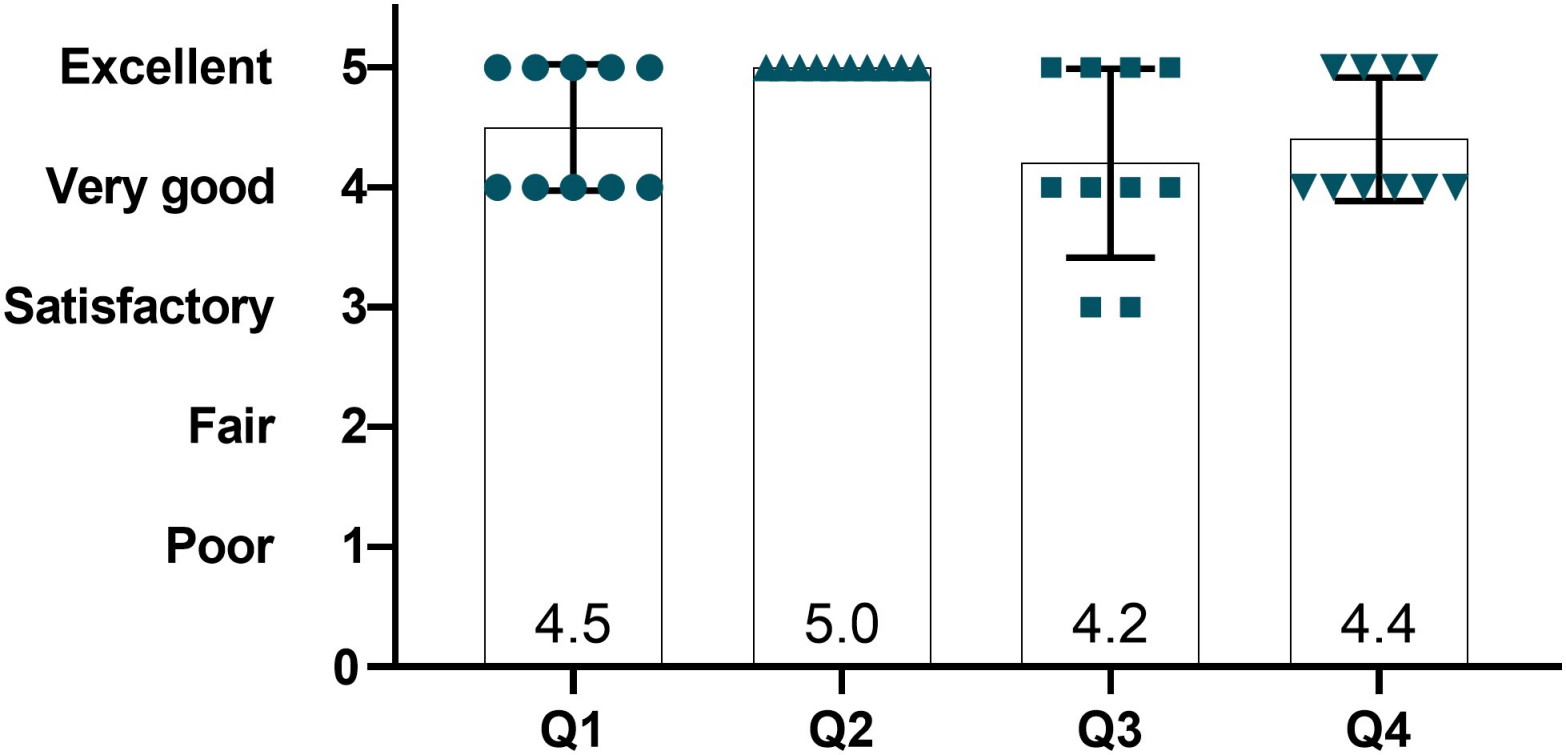
Surgeons assessment of model fidelity. Q1: Rate anatomical accuracy. Q2: Rate model size accuracy. Q3: Rate Landmarks visibility. Q4: Rate tactile fidelity during surgery (Haptic feedback). Data presented with mean and SD.

**Fig 5:**
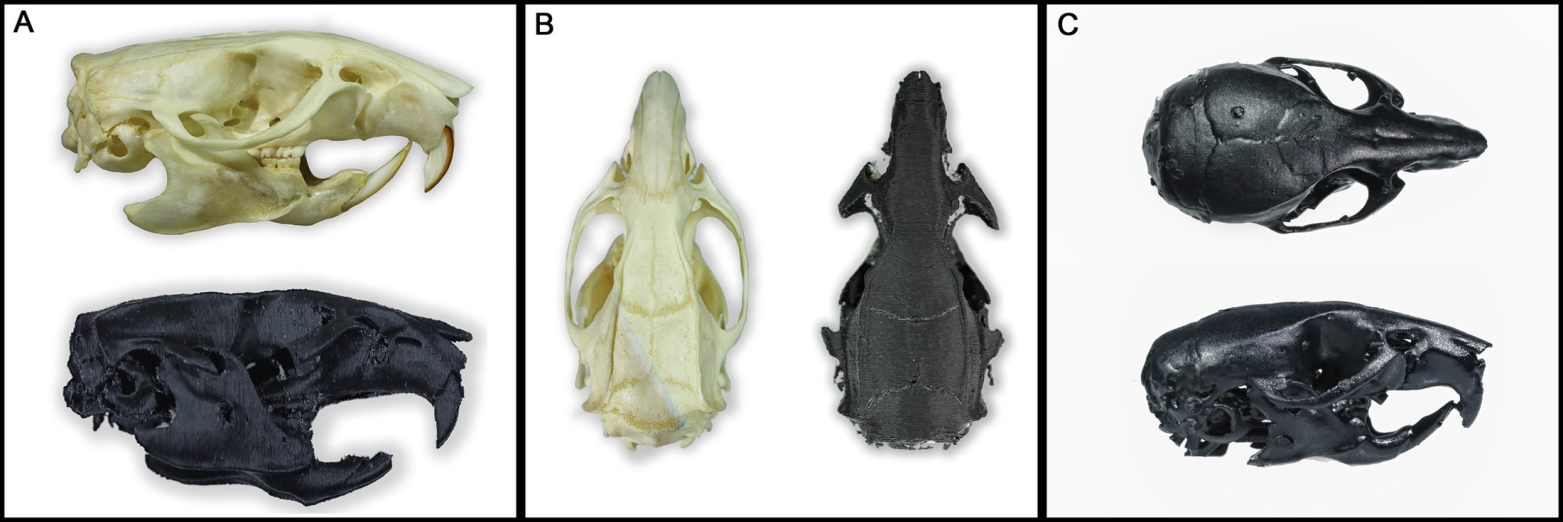
3D printed model rendering. (A) Comparison of 250g wistar rat skull and 3D printed model, Lateral view. (B) Comparison of 250g wistar rat skull and 3D printed model, Dorsal view. (C) Mouse 3D printed model, lateral and dorsal view.

The feedback from ten experienced neurosurgeons revealed high satisfaction (Fig 4) with very good anatomical accuracy (4.5). Both models show a very detailed real-size (5) replica of actual rodent skulls anatomy (Fig 5). Bone sutures forming Bregma and Lambda landmarks are identifiable (4.2). The 3D printed rodent models were perceived as very realistic, and even if the model did not perfectly replicate the feeling of real-animal brain surgery, tactile fidelity was positively rated (4.4).

**Fig 6:**
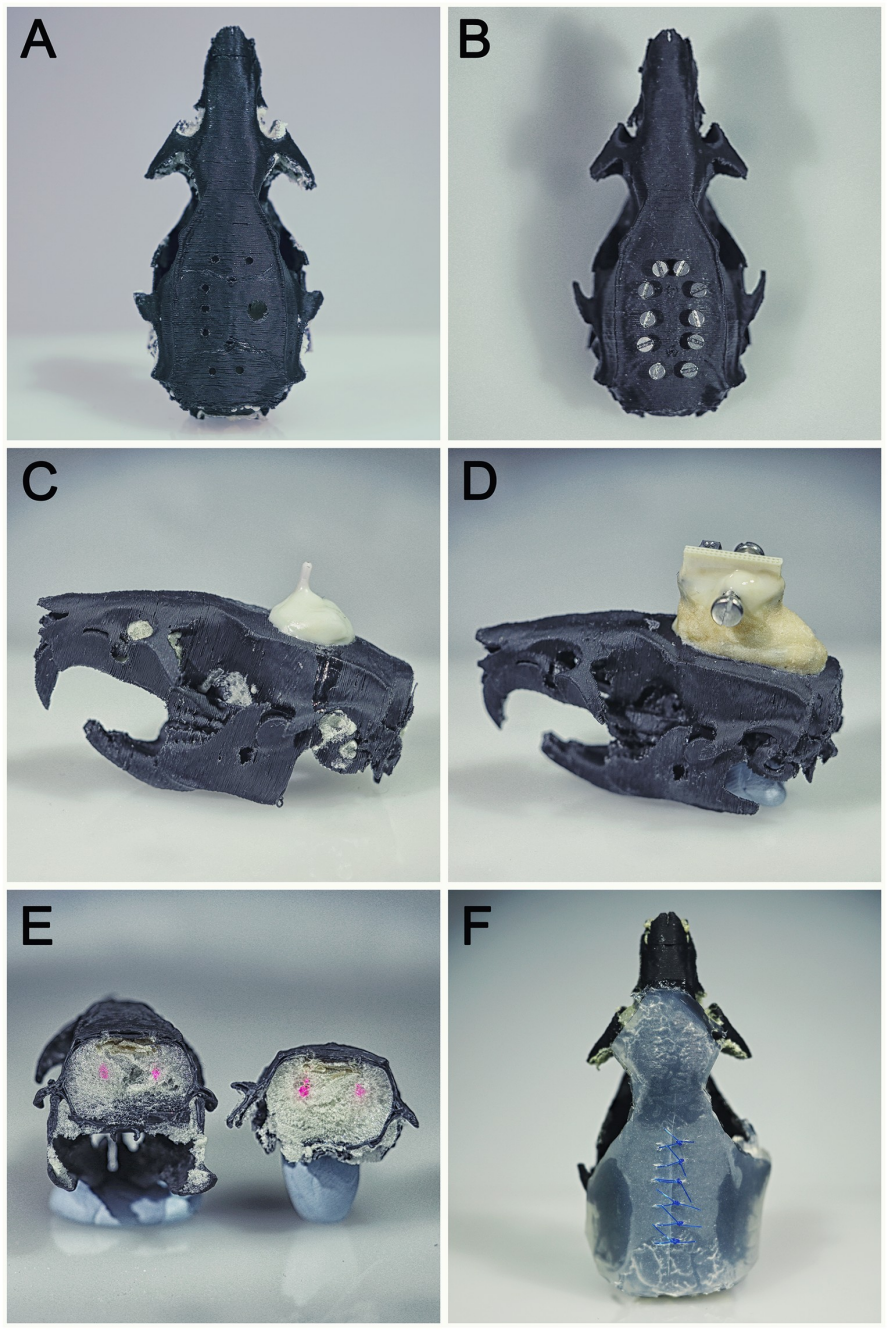
Different techniques that can be learned and practiced using the skin-skull-brain model. (A) Craniotomies. (B) Screws placement. (C) Optic fiber implantation and securing using bone/dental cement. (D) Large Electrophysiology probes implantation and securing using bone/dental cement. (E) Brain injections. (F) Suturing.

A wide range of neurosurgery techniques were successfully tested using these models: craniotomies (Fig 6A), screws placement (Fig 6B), Optic fiber (Fig 6C) and large Electrophysiology probes (Fig 6D) implantation and securing using bone/dental cement, brain injections (Fig 6E) and suturing (Fig 6F).

**Fig 7:**
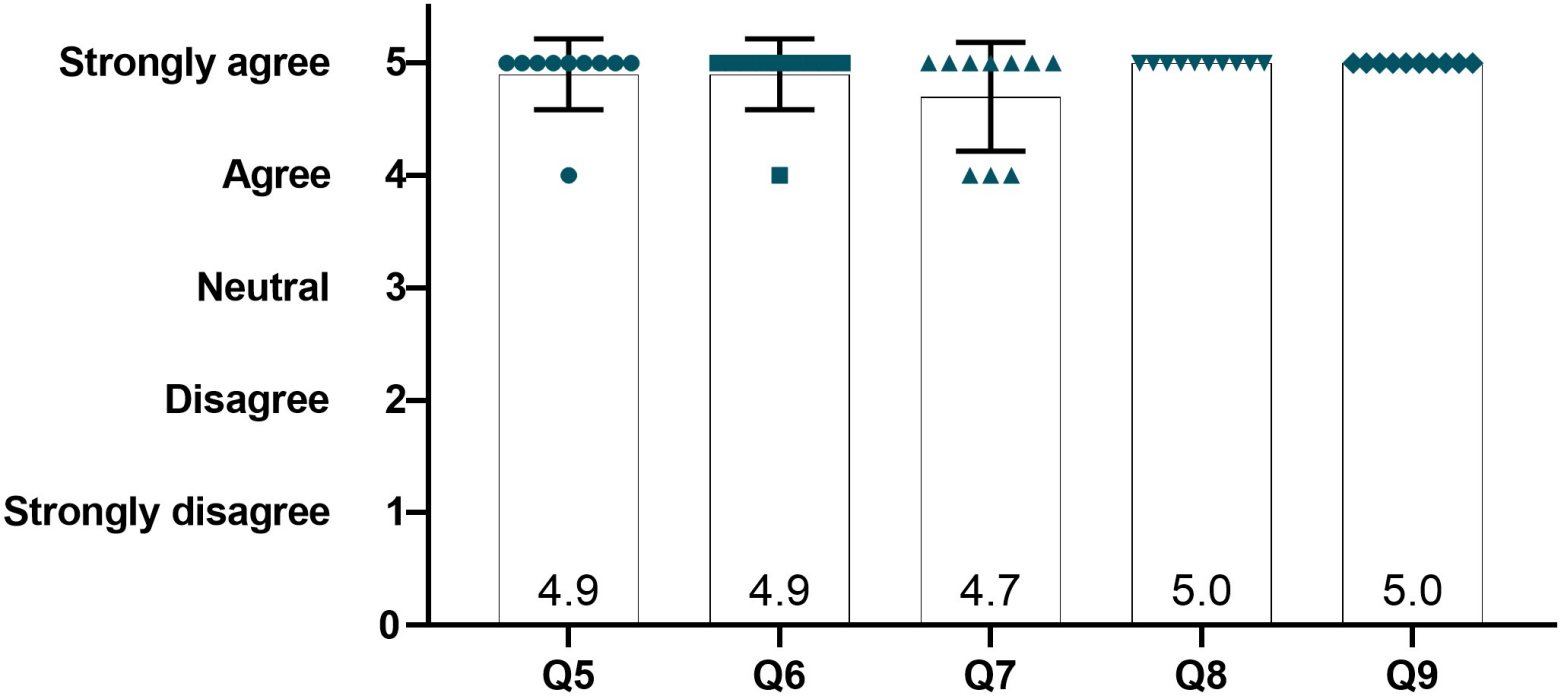
Surgeons assessment of appropriateness for educational use. Q5: Are these models appropriate and useful for surgery training? Q6: Are these models good alternatives to cadaveric skulls for training purposes? Q7: Would these models help trainees to develop their confidence? Q8: Would you use these models to train someone or to be trained on new techniques? Q9: Would you use these models to test the feasibility of new surgeries? Data presented with mean and SD.

Our models were reported to be appropriate and very useful for surgical training applications (4.9) as well as good alternatives to cadaveric skulls (4.9). They were stated to help trainees develop their confidence before working on live animals (4.7). All surgeons responded that they would actively use the model to train someone or to be trained on new techniques and to test the feasibility of new surgeries (5) (Fig 7).

## Discussion

Within the last decade, 3D printing technologies have penetrated and improved several fields of human healthcare from ophthalmology [18] to cardiovascular diseases [19] and neurosurgery [20], amongst others. 3D printing applications are now widely used for multiple purposes: pre-operative planning, teaching and training of naïve surgeons, counselling of patients before a surgery, and creating patient-specific implantable devices [21, 22].

3D printing use in neurosurgery is becoming more widespread with the creation of patient-specific anatomical models (e.g. 3D printed hollow models of cerebral aneurysms [23]). Such models provide an excellent tool for practice and rehearsal of the surgery by reproducing the patient exact anatomy and pathology.

Literature implies that rapid prototyping is well suited for the development of realistic 3D printed models that can be used for preoperative planning and surgery training [23–26] without putting patients at risk [21, 27].

Since the emergence of 3D printing technologies, cutting-edge research efforts are ongoing to develop and share 3D structures of vertebrates for education and research. Indeed, rat [8] and mouse [9] skull designs used here were shared by Bernd M. Pohl, Timothy Rowe and their colleagues in this spirit. To the best of our knowledge, 3D technology has never been translated to animal research in neuroscience for surgery training purposes.

The 3D printed skin-skull-brain models described here are high-fidelity and real-size replicas of rat and mice skulls. These 3D printed models are rapidly made (8 hours of unsupervised printing for the rat model and 2 hours for the mouse model) and low-cost (the raw materials cost 21 and 5 CHF, respectively).

Though 3D-printing materials are known for their inability to properly mimic soft biological tissues such as skin tissues, the recent development of a new technique called freeform 3D printing has revolutionized the field by allowing soft matters such as hydrogels and silicone elastomers to be printed [28]. The use of such techniques could improve our skin model; they would likely make the skin addition on our models more time-efficient, and by opening the door to other soft materials, they could provide an improved haptic representation of living animal skin, particularly useful for sutures.

One major advantage of our models is that 3D replicates are available in numbers and at any time enabling animal researchers to overcome the important limitations of numbers and availability of cadaveric animals. These models are very versatile as they offer training opportunities for many types of standard neurosurgeries techniques including: skin incision, craniotomies, screw placement, brain injections, long-term implantations and suturing. Moreover, these models may enable trainees to familiarize themselves with the many overwhelming new techniques they have to combine to perform neurosurgeries: the use of surgery tools, working under operating microscopes, performing in sterile conditions, thus creating an immersive and effective learning environment that has been shown as a highly effective learning environment [29].

Since our introduction of these models into our laboratories, they have already been used to test the feasibility of new surgeries (personal communication). The models proved especially useful in determining whether several probes would fit in a particular area, whether probes could be secured without hampering each other and whether an implant would fit in our MRI machine.

In 2018 and 2019 respectively, 886 and 788 people were registered as new animal experimenters in Switzerland (Unpublished data from Swiss Federal Food Safety and Veterinary Office). All of them are allowed to perform surgeries. In light of these numbers, it seems important to offer animal-free training options, and the models described here address all aspects of 3Rs.

These models are refining the use of laboratory animals as it changes the way to train people and ensure the best conditions for animals that undergo surgeries. By providing affordable training materials available at any time during the learning curve, we hope that these models will allow scientists, technicians and veterinarians to enhance their skills by practicing stereotactic techniques multiple times until they become fully efficient and reach a high success rate. By extensive training, surgery time could be shorter causing less anesthesia time, resulting in less animal distress during the procedure and allowing a better and faster post-surgery recovery.

Refining surgery skills should also increase the longevity of implanted devices with the potential to reuse animals for repeated non-painful studies. Moreover, risks of infections, misplacement of implants may also decrease.

We see this project as a major animal welfare step forward that could significantly reduce the use of animals for brain surgery training. These skin-skull-brain models provide an absolute replacement of both rats and mice for all types of brain surgery training.

## Supporting information

S1 Raw data Fig 4: Surgeons assessment of model fidelity. (XLSX)

S2 Raw data Fig 5: 3D printed model rendering. (XLSX)
